# Collective Cell Radial Ordered Migration in Spatial Confinement

**DOI:** 10.1002/advs.202307487

**Published:** 2024-03-23

**Authors:** Hao Dong, Fen Hu, Xuehe Ma, Jianyu Yang, Leiting Pan, Jingjun Xu

**Affiliations:** ^1^ The Key Laboratory of Weak‐Light Nonlinear Photonics of Education Ministry School of Physics and TEDA Institute of Applied Physics Nankai University Tianjin 300071 China; ^2^ State Key Laboratory of Medicinal Chemical Biology Frontiers Science Center for Cell Responses College of Life Sciences Nankai University Tianjin 300071 China; ^3^ Shenzhen Research Institute of Nankai University Shenzhen Guangdong 518083 China; ^4^ Collaborative Innovation Center of Extreme Optics Shanxi University Taiyuan Shanxi 030006 China

**Keywords:** active matter system, bottom‐up, collective cell migration, top‐down, 2D spatial confinement

## Abstract

Collective cells, a typical active matter system, exhibit complex coordinated behaviors fundamental for various developmental and physiological processes. The present work discovers a collective radial ordered migration behavior of NIH3T3 fibroblasts that depends on persistent top‐down regulation with 2D spatial confinement. Remarkably, individual cells move in a weak‐oriented, diffusive‐like rather than strong‐oriented ballistic manner. Despite this, the collective movement is spatiotemporal heterogeneous and radial ordering at supracellular scale, manifesting as a radial ordered wavefront originated from the boundary and propagated toward the center of pattern. Combining bottom‐up cell‐to‐extracellular matrix (ECM) interaction strategy, numerical simulations based on a developed mechanical model well reproduce and explain above observations. The model further predicts the independence of geometric features on this ordering behavior, which is validated by experiments. These results together indicate such radial ordered collective migration is ascribed to the couple of top‐down regulation with spatial restriction and bottom‐up cellular endogenous nature.

## Graphical abstract

Dong et al. reveal a collective ordered migration in 2D confined NIH3T3 fibroblasts, during which single cells move in a weak‐oriented diffusive‐like manner. The supracellular migration exhibits spatiotemporal heterogeneous and radial ordering, reflected by a radial‐ordered wavefront. Such coordinated behavior arises from the couple of top‐down regulation with spatial restriction and bottom‐up cellular endogenous nature.

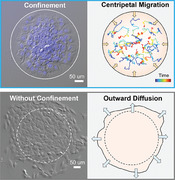

## 1 Introduction

In recent years, active matter systems^[^ ^1^, ^2^, ^3^, ^4^ ^]^ that contain large amounts of active units, from animal groups^[^ ^5^, ^6^, ^7^ ^]^ to bacteria^[^ ^8^, ^9^ ^]^ to functionalized colloids,^[^ ^10^, ^11^, ^12^ ^]^ have attracted a great deal of attention and became one of the most cutting‐edge topics, due to their intricate and mystery coordinated behaviors. Collective cells are an important class of active matter systems.^[^ ^4^, ^13^, ^14^ ^]^ Through cell self‐propulsion, cell‐cell coupling, and chemical reactions, diverse fascinating self‐organizing behaviors of collective cells emerge from the bottom‐up cross multi‐spatial and temporal scales, such as pattern formation,^[^ ^15^, ^16^ ^]^ phase separation,^[^ ^17^ ^]^ concerted migration,^[^ ^18^, ^19^, ^20^ ^]^ turbulent flow.^[^ ^21^ ^]^ These coordinated collective cell behaviors are essential for various momentous biological processes including embryogenesis,^[^ ^22^, ^23^ ^]^ morphogenesis,^[^ ^24^ ^]^ and tumor growth.^[^ ^25^ ^]^

Since all developmental and physiological processes take place in limited spaces or microenvironments, it is of great interest to study collective cell behaviors under confinement. Spatial restriction, a strategy that regulating cells from the top down, has been recognized to profoundly influence their spatiotemporal dynamical evolution, eventually determine cell fates and behaviors.^[^ ^26^, ^27^, ^28^ ^]^ Nowadays, emerging experimental techniques represented by cell micropatterning have been prevalently applied in offering precise spatial control.^[^ ^29^, ^30^, ^31^, ^32^, ^33^, ^34^, ^35^, ^36^ ^]^ Utilizing such top‐down strategy, combined with the intrinsic bottom‐up mechanism of multi‐cells, enables diversified ordering behaviors at supracellular scale, for instance, collective rotation^[^ ^31^, ^32^, ^33^ ^]^ and radial oscillations^[^ ^33^ ^]^ in circular constraints, wavelike oscillation in quasi‐1D channel,^[^ ^34^ ^]^ chiral morphogenesis on ring patterns,^[^ ^35^ ^]^ and directing tissue arrangement on parallel stripes.^[^ ^36^ ^]^ These findings largely enhance our understanding of the coordinated behaviors in active matter systems. However, because life activities are complicated and full of mysteries, more undiscovered collective cell phenomena within spatial constraints remain to be further explored. By revealing the emergence and evolution of these dynamical behaviors, new frameworks for investigation of these systems can be built.

In this study, we report a centripetal migration behavior of NIH3T3 fibroblasts in 2D geometric confinement by cell micropatterning. To understand the characteristics and mechanisms of this process, we analyze the spatiotemporal dynamics of single and collective cells, and investigate the effects of different restriction sizes and shapes. Numerical simulations employing a developed mechanical model with spatial confinement were further executed to explain experimental observations. Results underline the collective cell radial ordered migration is ascribed to the combination of top‐down regulation with spatial constraints and bottom‐up cellular endogenous property.

## 2 Results

### 2.1 Collective Centripetal Migration of NIH3T3 Fibroblasts Confined to 2‐D Circular Substrates

We start with fabricating micropatterned substrates utilizing photolithography technique as described in our previous work.^[^ ^37^ ^]^ The micropatterns consisted of adhesive, fibronectin‐functionalized areas with different sizes and shapes, separated by non‐adhesive barriers passivated with poly‐l‐lysine‐graft‐PEG (PLL‐PEG, 200 µg mL^−1^ for 1 h), a commonly used protein‐repelling regent. NIH3T3 cells were plated into the circle patterns with a diameter of 400 µm, and treated with mitomycin to block cell proliferation.

**Figure 1:**
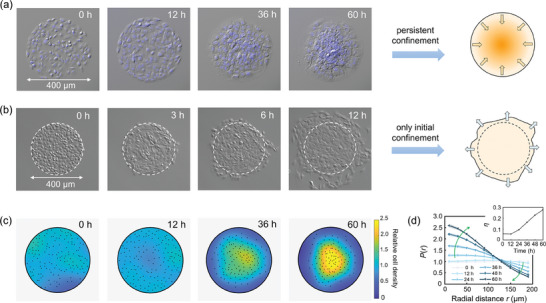
The collective migration of NIH3T3 cells in circular confinement. a) Time evolution of NIH3T3 fibroblasts persistent confined to circular patterns with a diameter of 400 µm. The nuclei were labeled by Hoechst 33342 (blue). b) Evolution of cells constrained only at the initial phase. The dashed white line indicates initial boundary. c) Heat maps of relative cell density *p*(**r**) evaluated by a modified KDE algorithm. The black dots represent the mass center of each nucleus, corresponding to (a). (d) Radial profiles of relative cell density *P*(*r*) at 0, 12, 24, 36, 48, 60 h (n = 7). Data are summarized from at least three independent experiments, with mean and standard error of mean (SEM) indicated by solid lines and shaded regions, respectively. Inset: evolutions of cell density heterogeneity *η* (n = 7, mean ± SEM).

Interestingly, we observed an endocentric migration phenomenon of NIH3T3 cells in the circular confining substrate (**Figure** **1**a; Movie S1, Supporting Information). Without external photo‐, electro‐, duro‐ and chemotaxis, collective cells spontaneously converged to the center of pattern from all directions, independent on cell proliferation. In contrast, cells that were only constrained at the initial phase (by low concentration PLL‐PEG, 2 µg mL^−1^ for 1 h) would expand outward the boundary (Figure 1b), indicating that persistent 2‐D space restriction is indispensable for the collective cell aggregation. Besides, similar centripetal migration behavior of NIH3T3 cells was observed in different circular sizes (diameter = 600, 800, 1200 µm), whereas larger sizes with less aggregation extent (Figures S1 and S2 and Movie S2, Supporting Information). Therefore, the top‐down regulation with restriction per se determines the occurrence of collective cell centripetal migration, regardless of specific geometric size.

To quantify the degree of aggregation, we evaluated the distribution of relative cell density *p*(**r**)by a modified Kernel density estimation algorithm, where **r** represents the spatial position. The evolution of *p*(**r**) was then visualized by heat maps (Figure 1c), showing that cell density progressively decreased at the boundary and increased at the center. We further azimuthally collapsed the heat maps with a ring‐width of 20 µm to obtain radial profiles of cell density,

(1)

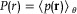

(Figure 1d), where *r* represents radial distance to the center of the circle. It was found that the relative cell density declines with the radial distance after 24 h, with the decline rate rising over time. Next, the cell density heterogeneity was characterized by *η*, defined as,

(2) $$\eta =\frac{1}{2S}\underset{S}{\;\int \int \;}\left|p(r)-1\right|dr$$

where *S* represents the area of the pattern. *η* ∈ [0, 1], the closer the value is to 0 (1), the more homogeneous (centralized) the system is. As shown, *η* exhibited a monotonically increase with time from nearly 0 to 0.276 ± 0.007 (mean ± SEM) (Figure 1d, inset). These results together suggest that confined collective cells time‐dependently concentrated to the pattern center, marked by transitions from homogeneous to heterogeneous states.

### 2.2 Single Cells Move in a Weak‐Oriented, Diffusive‐Like Rather Than Strong‐Oriented Ballistic Manner

**Figure 2:**
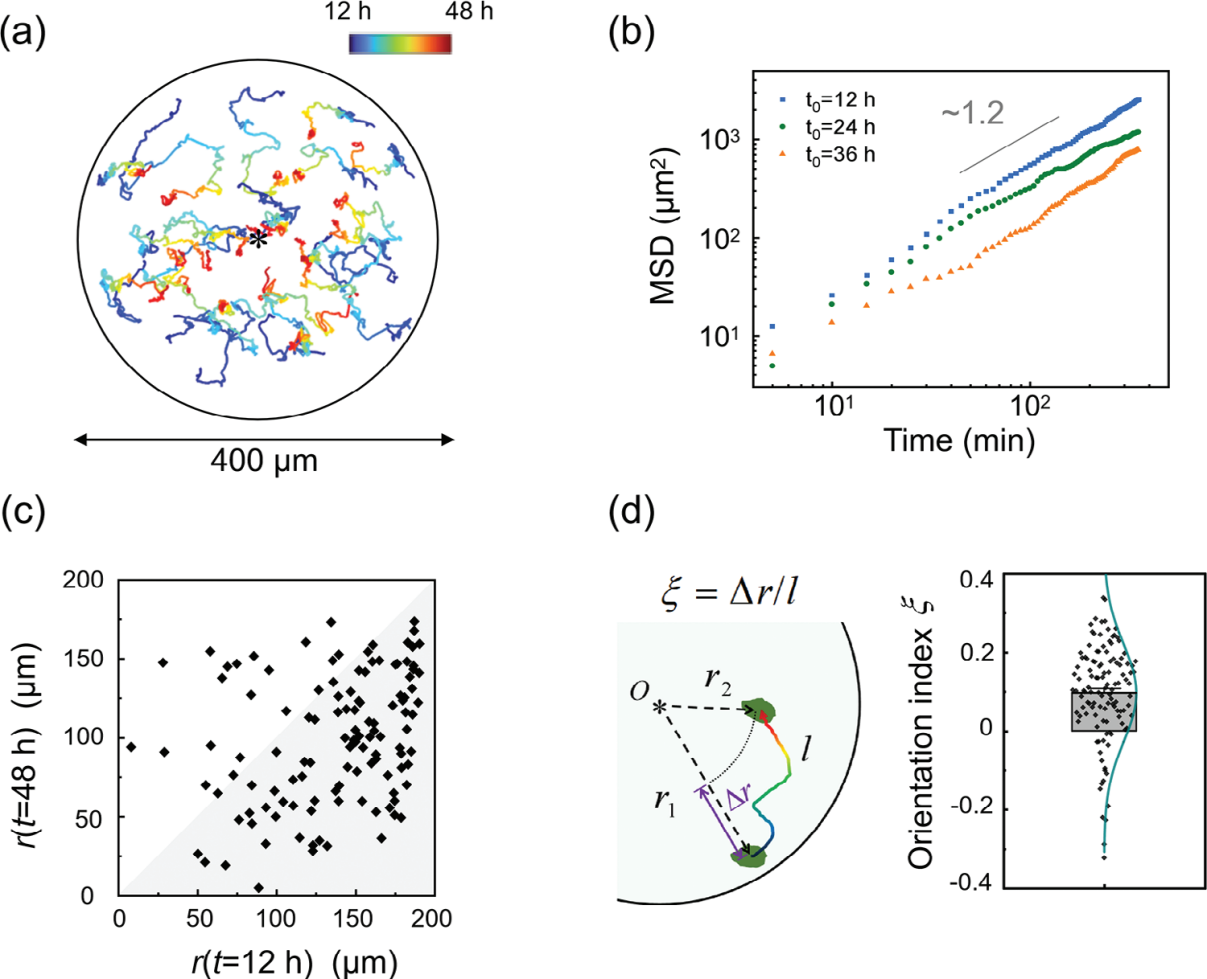
The motion characteristics of individual cells during the centripetal migration. a) Trajectories of representative 20 cells from 12 to 48 h, visualized in a time‐coded color representation, ranging from blue to red. The asterisk represents the center of the circle (diameter = 400 µm). b) MSDs of cells at different starting moments *t*_0_, at starting moments *t*_0_ = 12, 24, and 36 h, with a scaling exponent *α* = 1.25, 1.16, and 1.20, respectively, (n = 119). c) Scatter plot showing the relation of radial positions of the same cell at 12 h and 48 h. Pearson's correlation coefficient *ρ* = 0.30. Points in gray region represent cells that are closer to the center. d) Diagram of orientation index *ξ* (left) and its statistical distributions of cells (*ξ* = 0.095 ± 0.011, n = 119, mean ± SEM), fitted by a Gaussian profile (right).

Subsequently, individual cells were tracked manually from 12 to 48 h by ImageJ to analyze their motion characteristics during the centripetal migration process (Figure **2**a), quantified by mean square displacement (MSD):

(3) $$\mathrm{MSD}\left(t\right)=\frac{1}{n}\sum_{i=1}^{n}{\left[r_{i}\left(t\right)-r_{i}\left(t_{0}\right)\right]}^{2}$$

where *t*_0_ is the starting time point, and *n* is the number of selected cells. We calculated the MSDs of the cells for each 12 h interval (12‐24 h, 24–36 h, and 36–48 h) (Figure 2b). The MSDs were then fitted by Power‐law functions *Kt^α^*, where the scaling exponent *α* = 1 represents random diffusive motion, and *α* = 2 represents persistent ballistic motion. For the three time periods, we derived *α* = 1.25, 1.16, and 1.20 respectively (Figure 2b), suggesting that the locomotion of individual cells appears to be more random than ballistic.

By analyzing the variations of radial position of representative cells, we next found that although most (81.5%) of cells migrate toward the center, there is still a small proportion moved away from the center (Figure 2c). There is no significant correlation between radial positions pre‐ and post‐aggregation (Pearson correlation coefficient = 0.30) (Figure 2c), implying a fluctuate orientation of single cells via intercellular position rearrangement. To quantify the migration directionality, we defined orientation index *ξ* = Δ*r*/*l* in Figure 2d, where

(4) Δr=r(t=48h)−r(t=12h)

*l* is the corresponding route. *ξ* ∈ [‐1, 1], a value closer to 1 indicates more oriented to center, −1 does the opposite. Results showed that *ξ*= 0.095 ± 0.011 for this centripetal migration (Figure 2d), indicating NIH3T3 cells are weakly directed to the center. In contrast to our results, the system of ADP‐induced microglia chemotaxis, an acknowledged directed migration process, derived *α* = 1.83 and ξ= 0.658 ± 0.047 respectively (Figure S3 and Movie S3, Supporting Information), exhibiting a strong‐oriented ballistic‐like motion characteristic. Hence, within 2‐D constraints, single cells migrated to the pattern center weak‐oriented diffusively instead of strong‐oriented ballistically.

### 2.3 Spatiotemporal Heterogeneous and Radial Ordering of the Collective Cell Migration

**Figure 3:**
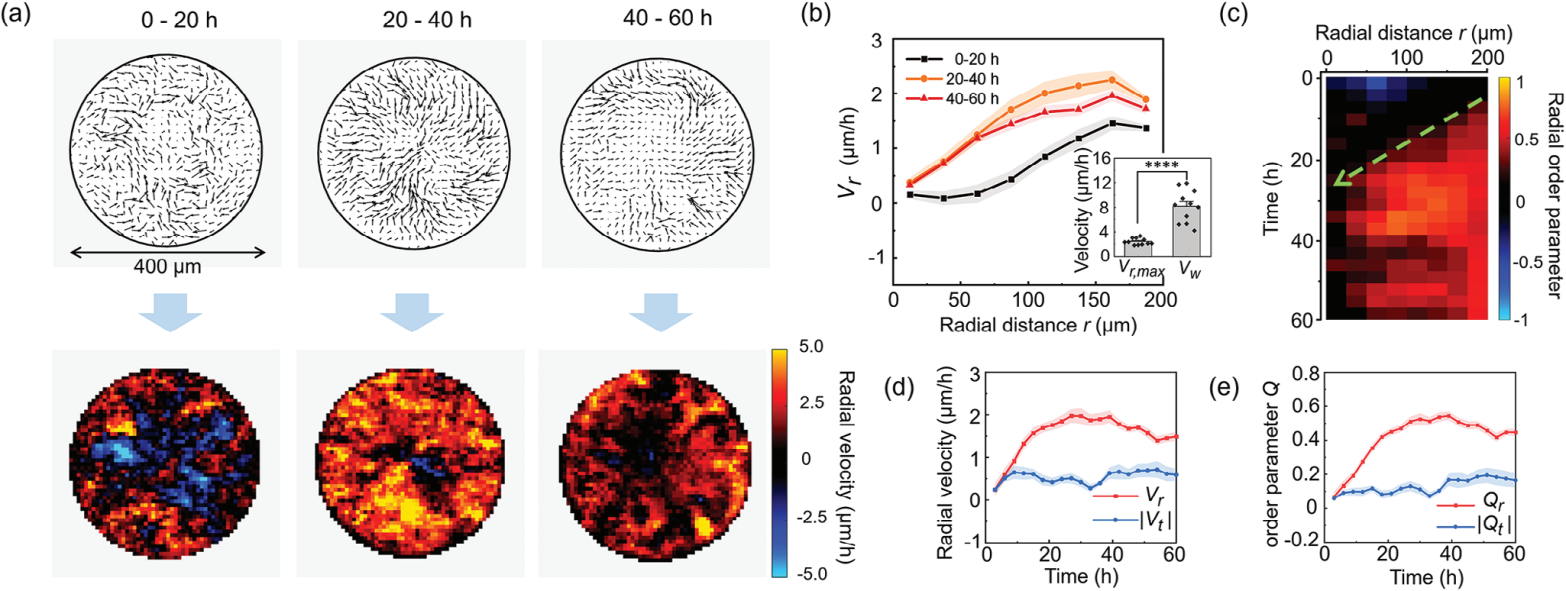
Spatiotemporal dynamics of collective cell centripetal migration. a) Average velocity fields for *T* = 0–20 h, 20–40 h, and 40–60 h derived by PIV analysis (up). Heat maps of the radial component of the average velocity fields (down). b) Profiles of the radial velocity *V_r_* at *T* = 0‐20 h, 20–40 h, and 40–60 h (n = 11, mean ± SEM). Inset: Comparison between the maximum radial velocity *V*_*r*,max _ and the speed of wavefront *V_w_* (n = 11, mean ± SEM, ^****^ *p* < 0.0001, paired *t*‐test). c) Kymograph of the radial order parameter. The dashed green line indicates a split line fitted with a threshold *q* = 0.20. d) Time evolution of average radial velocities *V_r_* and average tangential average |*V_t_* | (absolute values are taken for both tangential directions) for collective cells in the entire pattern (n = 11, mean ± SEM). e) Time evolution of radial order parameter *Q_r_* and tangential order parameter |*Q_t_* | for collective cells in the entire pattern (n = 11, mean ± SEM).

Furthermore, particle imaging velocimetry (PIV) method was applied for automatically calculating the cellular velocity field **v**(**r**, *t*) with a spatial resolution of 16 µm and a time resolution of 5 min. Due to the weak orientation of individual cells, we computed the average velocity field **v**_*m*_(**r**,*T*) = 〈**v**(**r**, *t*)〉_*T*_ for three durations *T* = 0–20 h, 20–40 h, and 40–60 h respectively (Figure **3**a, up). It manifested a predominant orientation of cells pointing to the center at *T* = 20–40 h and 40–60 h, implying a radial ordering of this process at supracellular scale. Then, we plotted a heatmap of the radial component of the average velocity field *v_r_* (**r**,*T*) = [**v**_*m*_(**r**,*T*)]_*r*_ (Figure 3a, down), and its radial profile *V_r_* (*r*,*T*) = 〈*v_r_* (**r**,*T*)〉_θ_ (Figure 3b). As shown, cells near the boundary initiated the migration toward the center (Figure 3a, *T* = 0‐20 h), followed by global centripetal migration of cells in almost the entire pattern (Figure 3a, *T* = 20‐40 h, 40–60 h), indicating the confined cells successively converged to the center, rather than simultaneously. Besides, it is noteworthy that the mean radial velocity near the boundary was much greater than that at the center (Figure 3b).

To further explore this spatiotemporal heterogeneous, we defined radial order parameter 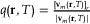, and obtained its kymograph for visualizing the spatiotemporal evolution over 60 h with an average time‐period Δ*T* = 6 h (Figure 3c). Results showed an increase in radial ordering at the pattern boundary, which spread toward the center like a wavefront (green dotted line in Figure 3c). Strikingly, the speed of this radial ordered wavefront *V_w_* significantly exceeds the maximal radial velocity *V*_*r*,max _ = max [*V_r_* (*r*,*T*)] (8.2 ± 0.8 µm h^−1^ vs 2.4 ± 0.2 µm h^−1^) (Figure 3b, inset), implicating this faster radial ordered wavefront may serve as a long‐range signal transmission mechanism at supracellular level to lead the way of cell locomotion toward the center, eventually forming global cell centripetal migration.

Moreover, in comparison with the radial component of average velocity (*V_r_, V_r_* = 〈[**v**_m_(**r**)]_*r*_〉_*S*_), the tangential component (*V_t_,V_t_* = 〈[**v**_*m*_(**r**)]_*t*_〉_*S*_) can be almost ignored (Figure 3d). Similarly, the average radial order parameter*Q_r_* = 〈*q*(**r**, *T*)〉_*S*_ (where〈〉_*S*_ denote averaging over the entire pattern), is much greater than tangential order parameter 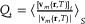 (nearly zero at each time course) (Figure 3e), demonstrating the radial orderliness of collective cell centripetal migration at the multicellular level. Additionally, we observed akin radial ordered wavefront in larger sizes of circular pattern (diameter = 600, 800, and 1200 µm) (Figure S4a–c, Supporting Information). The radial velocity curves exhibited comparable decline tendency, especially with nearly overlapping decaying from the boundary to the interior at 0–20 h (Figure S4d, Supporting Information), underlining the restriction boundary is a key decisive factor for the initial migration state, independent on the sizes of pattern.

### 2.4 Simulations of the Collective Cell Centripetal Migration Based on a Developed Mechanical Model in 2‐D Spatial Constraint

**Figure 4:**
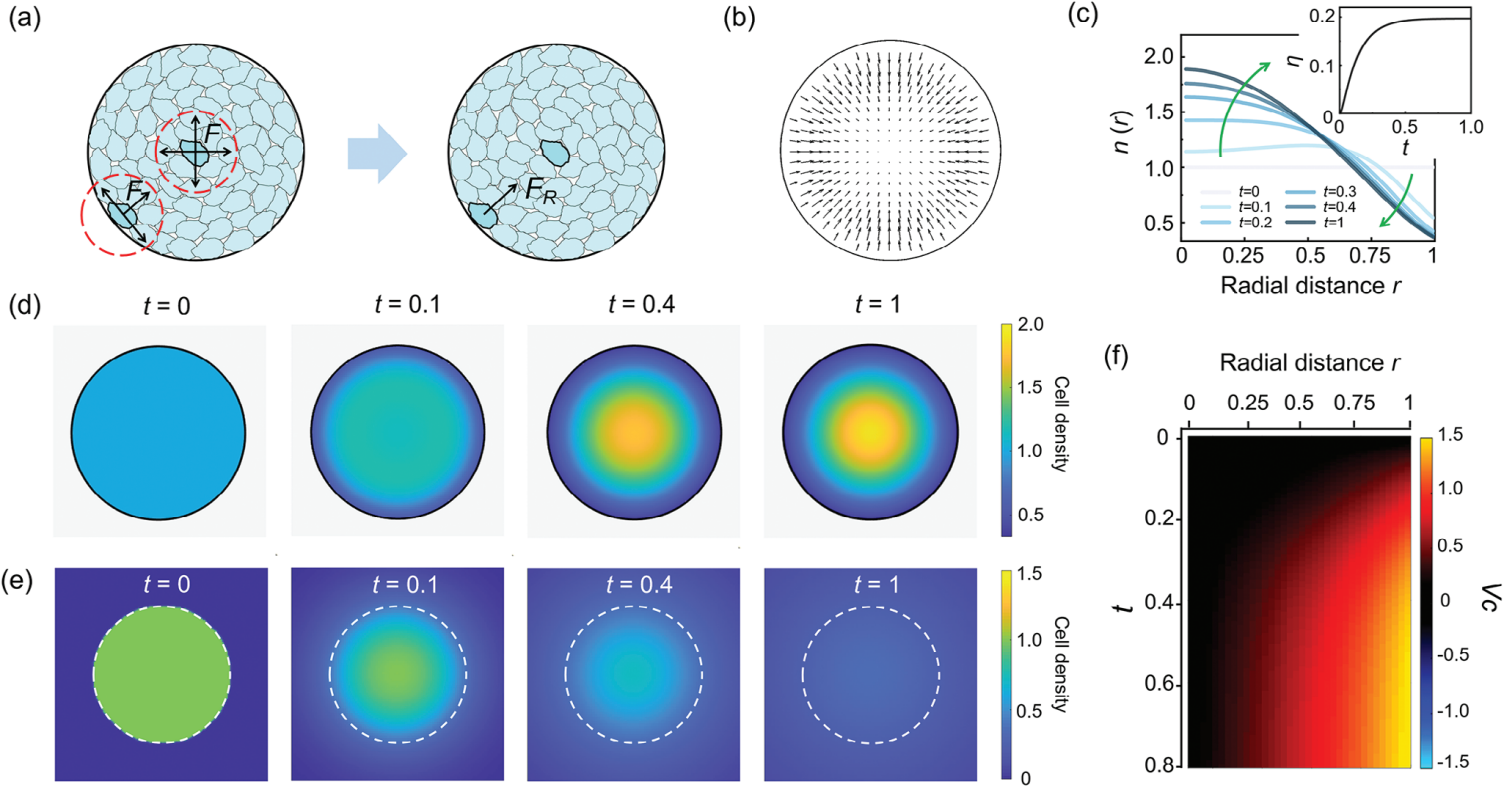
Simulations of collective cell centripetal migration in circular restriction. a) A diagram illustrating the forces on cells near the boundary and the center. ***F***, the ECM tensile force from their surrounding cells; ***F_R_***, resultant force of ***F***. b) Representative cellular velocity field **V**(**r**, *t*) maps. c) Simulated radial cell density profiles *n*(*r*). Inset: simulated evolutions of cell density heterogeneity *η*. d) Simulated evolution of cell density distribution *n*(**r**, *t*). e) Simulated evolution of *n*(**r**, *t*) when spatial constraint was removed at *t* >0 with other parameters kept constant and equal to (d). (f) Kymograph of the radial velocity *V*_**c**_(*r*). (*α* = 1.8, *s* = 16, *L_µ_* = 1, and *R* = 1, where *R* is radius of circular pattern).

It is well‐known that fibroblasts secrete abundant extracellular matrix (ECM） proteins,^[^ ^38^, ^39^ ^]^ thus the bottom‐up endogenous cell‐to‐ECM interactions may be critical for collective cell behaviors. Accordingly, we develop a simplified mechanical model in 2‐D spatial constraint, which emphasizes the force arising from cell‐to‐ECM interactions. In the model, we make the following assumptions: 1) Cells execute diffusion‐like movement within a confined region; 2) Cells exert tensile force on their surrounding cells by secreting ECM proteins, which degrade over time; 3) Cells move along the direction of the resultant tensile force, which is the sum of tensile force caused by all the surrounding cells through ECM (**Figure** **4**a) (See more details in Experimental Section and Supporting Information). This type of guided migration is called “plithotaxis” according to previous literatures.^[^ ^40^, ^41^ ^]^

Based on above hypothesis, for the cells in the center, the ECM force from their surrounding cells is symmetric, so the resultant force is 0. However, for the cells near the boundary, the symmetry breaking of cell distribution results in asymmetric force, thus establishing an inward force field (Figure 4a). Furthermore, such force field gives rise to an inward velocity component **V**_**c**_, which overwhelms the outward velocity component **V**_**d**_ due to hinderance of boundary diffusion within confinement. Thus, the superposed velocity field **V** = **V**_*c*_ + **V**_*d*_ is inward (Figure 4b; Figure S5a, Supporting Information). This inward velocity field leads to time‐dependent transition from homogeneous to heterogeneous states of cell density distribution, as shown by radial profiles of cell density *n*(*r*) and time evolution of *η* (Figure 4c), as well as the heatmaps of cell density distributions *n*(**r**, *t*) (Figure 4d; Movie S4, Supporting Information). On the contrary, simulation indicates that when cells were constrained only at the initial phase, diffusion would instead play a dominant role, resulting in cell spreading outward (Figure 4e; Figure S5b, Supporting Information), which agrees with the experiments.

Besides, kymographs of **V**_**c**_(*r*) from the simulation (Figure 4f) show akin patterns to that from experiment (Figure 3c), revealing that the generation of ordered wavefront may be resulted from the establishment of inward force field. Simulations also show collective cell convergence in other sizes of circular pattern, with reduced degree in larger sizes (Figure S6c,d, Supporting Information), consistent with experimental results (Figure S2, Supporting Information). Taken together, by introducing bottom‐up ECM‐associated plithotaxis, the experimental observations in confinement can be well reproduced and explained by simulations. Also, our model elucidates how the bottom‐up internal mechanisms coupled with the top‐down regulation by spatial constraints.

### 2.5 The Collective Cell Centripetal Migration is Independent on the Geometric Features of 2‐D Restriction

**Figure 5:**
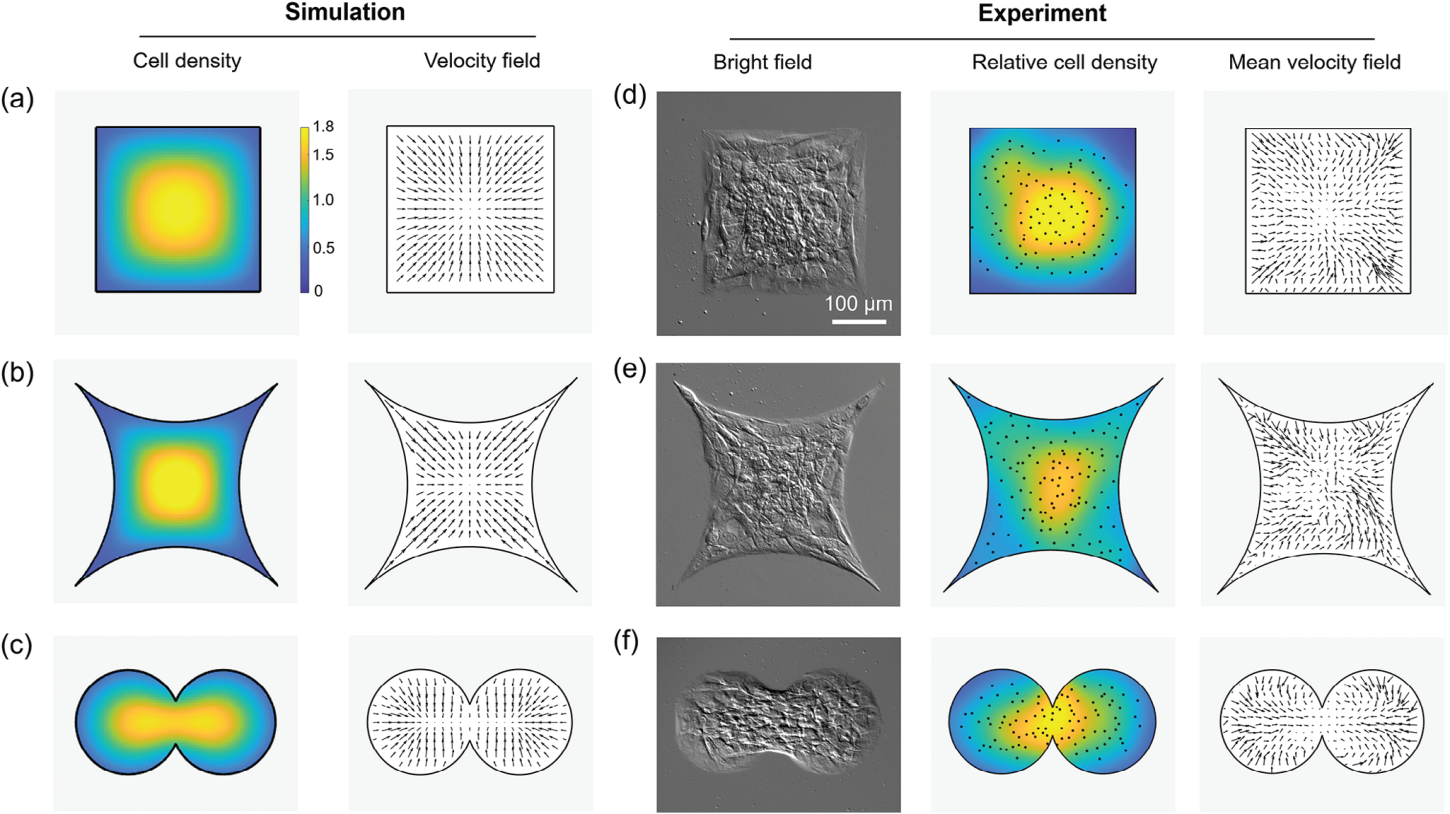
Simulation and experiment results of collective cell confined in pattern shapes. a–c) Simulations of cell density heat maps and representative cellular velocity field maps for square (a), indented‐square (b), and peanut‐like patterns (c). (*α* = 1.8, *s* = 16, *L_µ_* = 1, *d* = 1.5, *R_p_* = 1.5, *R_o_* = 0.5, and *d_o_* = 0.07, where *d* is side length of the square pattern, *R_p_* is negative radius of indented‐square boundary in indented‐square pattern, *R_o_* and *d_o_* is the radius of each circle and the overlap distance between two circles in the peanut‐like patterns, respectively). d–f) The bright field images, relative cell density heat maps, and average velocity field maps (average 10–50 h) of collective cells in different pattern shapes including square with side length of 300 µm (d), indented‐square with a negative radius of curvature of 300 µm (e), and overlapping circle with diameter of 200 um with an overlap distance of 7 µm (f). Cells were cultured for ∼60 h. Black dots in the hot maps represent the central position of nuclei.

Although our simulation results match all the experimental observations in the circle pattern with positive‐curvature boundary, we still wonder whether there was similar aggregation behavior in other pattern shapes. Based on our model, as long as a persistent spatial constraint is present, a superposed inward polarization field can be formed perpendicular to the restriction boundary to direct cell migration, no matter what geometric size and shape the constraint are. To address this, we simulated the collective cell behavior in different geometric constraint shapes including a square pattern with zero‐curvature boundary (**Figure** **5**a), and an indented‐square pattern with a negative‐curvature boundary (Figure 5b). Simulations showed similar centripetal migrations for both patterns, with the velocity field radiating toward the center. We also designed an interesting peanut‐like pattern. Counter‐intuitively, the cells congregated to the center of the whole pattern, instead of converging to the centers of the two circles separately (Figure 5c). Experiment data (Figure 5d‐f; Movie S5, Supporting Information) were well in accordance with these simulation results, further corroborating the reliability of our model.

## 3 Discussions and Conclusion

Spatial restriction is ubiquitous in biological systems, and plays an important top‐down guiding role in active matter systems such as collective cells. In different confining conditions, multiple ordering cell behaviors emerges, involving cell rotation,^[^ ^31^, ^32^, ^33^, ^42^, ^43^ ^]^ chiral arrangement,^[^ ^35^ ^]^ wave oscillations,^[^ ^34^ ^]^ sheet migration,^[^ ^30^ ^]^ etc. The emergence of these collective behaviors embodies complex interplay between cells and their surrounding environments. In the present study, utilizing top‐down regulation with 2‐D geometric confinement, we discover a spontaneous collective centripetal migration behavior of NIH3T3 fibroblasts in the absence of exogenous physicochemical stimuli, leading to cell aggregation (Figure 1a). Despite a weak‐oriented, diffusive‐like dynamics of single cells (Figure 2), the collective migration found here exhibits radial ordering at supracellular scale, as specifically shown by the emergence and propagation of a radial ordered wavefront from the boundary to pattern center (Figure 3). Withdrawing the spatial constraints terminated this ordered migration (Figure 1b), confirming the necessity of spatial restriction in inducing collective cell ordering behaviors.

However, it is puzzling that being similarly top‐down regulated by circular pattern restriction, such radial ordered collective migration of NIH3T3 is quite different from the extensively reported rotation occurred in MDCK epithelial cells.^[^ ^31^, ^32^, ^33^, ^42^, ^43^ ^]^ Because bottom‐up mechanisms such as intercellular adhesion,^[^ ^44^, ^45^ ^]^ and cell‐extracellular matrix interactions,^[^ ^46^, ^47^ ^]^ cellular chemotaxis,^[^ ^15^, ^16^, ^36^, ^46^ ^]^ also significantly affect cell behaviors, we speculate this distinct ordering state may originate from different bottom‐up endogenous properties. It has been established that the collective rotation of MDCK cells relies on sustained memory of cell polarity and strong cell‐to‐cell adhesion characteristics,^[^ ^32^, ^48^, ^49^ ^]^ whereas NIH3T3 cells show weak intercellular adhesion and interactions^[^ ^50^, ^51^ ^]^ (also proved by our results in Figure S7, Supporting Information). Taking a full consideration of cell‐to‐cell adhesion in collective cell behaviors, we run simulations by classic cell Potts model (CPM),^[^ ^52^, ^53^, ^54^ ^]^ and successfully mirror the collective rotation phenomenon of MDCK cells (Figure S8a–c, Supporting Information). In contrast, centripetal migration occurs under neither strong nor weak intercellular adhesion (Figure S8d–h, Supporting Information), ruling out the possibility of cell‐to‐cell adhesion in eliciting collective cell radial ordered migration.

On the other hand, cellular chemotaxis is also a common bottom‐up mechanism in leading directed cell migration, such as the aggregation of bacteria.^[^ ^55^ ^]^ Therefore, if NIH3T3 cells self‐secrete certain chemokines to form a centripetal chemotactic gradient, it may be a reasonable explanation for their collective movement. To test this, we stirred the culture medium every 2 h to disturb the potential chemotactic gradient. However, the centripetal movement was not affected by these treatments (Figure S9 and Movie S6, Supporting Information), excluding the possibility of such a mechanism. This is understandable because the chemotaxis mechanism is indeed very susceptible to be interfered or disrupted, and this is not adapted to the complex and changing microenvironment during physiological processes. Hence, the centripetal movement of NIH3T3 cells must be attributed to another, more stable mechanism.

It is well known that ECM provides principal means of intercellular mechanical communication,^[^ ^38^, ^56^ ^]^ thereby playing key roles in tissue formation and morphogenesis.^[^ ^57^, ^58^ ^]^ Furthermore, fibroblasts are a type of cells that abundantly produce, secrete, and remodel ECM proteins.^[^ ^38^, ^39^, ^59^ ^]^ Our simulations based on an ECM force‐associated mechanical model well reproduce the experimental results, suggesting the endogenous mechanism could be explained by ECM‐associated plithotaxis. It shows that in the presence of spatial constraints, due to the symmetry breaking of cell distribution, an inward force field mediated by ECM generates at the restriction boundary, directing cell migration toward the center (Figure 4a,b). Otherwise, if the bottom‐up ECM‐associated plithotaxis mechanism is excluded, the cells would be uniformly distributed within the entire pattern due to cell diffusion (Figure S10, Supporting Information). These modeling provide an explanation for how bottom‐up mechanisms combine with top‐down regulations to induce the ordered movement of collective cells. Besides, the possible involvement of ECM‐associated plithotaxis was further supported by the Ca^2+^‐free experiment, due to the dependence of cell‐ECM connection on Ca^2+[^ ^60^ ^]^. It showed an evident inhibition of collective cell centripetal movement after removal of extracellular Ca^2+^ (Figure S11 and Movie S7, Supporting Information). Despite this, we cannot exclude the possibility that other models with different bottom‐up mechanisms may predict similar results.

Furthermore, if there were no spatial constraints, what will happen only with bottom‐up cellular mechanisms? A previous work revealed that in free space, vascular mesenchymal cells (VMCs) could spontaneously aggregate to ridges in vitro, forming unordered labyrinthine Turing patterns.^[^ ^16^ ^]^ This phenomenon is explained by classical activator‐inhibitor reaction‐diffusion model. Interestingly, applicating top‐down regulation with micropatterned substrate, the ridges of VMCs exhibited an ordered directing alignment.^[^ ^36^, ^61^ ^]^ Similarly, in our results, after removal of spatial restriction, the bottom‐up ECM‐associated plithotaxis alone leads to spread outward of cells instead of inducing a radial ordered migration (Figure 4e; Figure S12, Supporting Information). Thus, top‐down regulation with spatial confinement plays pivotal roles in determining the orderliness of collective cell behaviors.

In addition, the radial ordered migration depends on the restriction per se, whereas irrespective of specific geometric sizes or shapes (Figure 5). In contrast, some constraints‐dependent ordered behaviors are directly influenced by the geometric features. For instance, cell rotation is more likely to occur in smaller circular patterns.^[^ ^48^, ^62^, ^63^ ^]^ Chiral morphogenesis appears in a ring pattern rather than in circular or square patterns.^[^ ^35^ ^]^ Sheet migration requires narrower bands, with wider ones resulting in swirls.^[^ ^30^ ^]^ The independence on geometric features of confinement is determined by the distinctive bottom‐up ECM‐associated plithotaxis mechanism, because the formation and maintenance of biased force field only needs top‐down regulation with restriction per se. This may also provide a novel insight for understanding the stability and adaptability of life activities in complicated and ever‐changing external environment at cellular level.

Overall, this work unveils a collective cell radial ordered migration of NIH3T3 fibroblasts in spatial confinement, and explained this distinctive migration behavior based on a developed mechanical model. Experimental and simulation results together indicate this collective migration is resulted from a combination of top‐down regulation with spatial restriction and bottom‐up endogenous cellular property. These findings will provide new perspectives in the research of coordinated behaviors in active systems under spatial confinement, as well as enhancing our understanding of how the different bottom‐up cellular mechanisms lead to the formation of diverse organizational structures during development. We anticipate that these insights may inspire design ideas for the fabrication of artificial tissues and organoids.

## 4 Experimental Section

### Cell Culture

NIH3T3 cells were cultured in Dulbecco's modified Eagle's medium (Gibco, USA) supplemented with 10% (v/v) fetal bovine serum (Biological Industries, USA) and 1% penicillin and streptomycin (Gibco, USA). Cells were maintained at 37 °C under humidified atmosphere (5% CO_2_).

### Preparation of Micropatterned Substrate

First, clean glass coverslips were deposited by a single molecular layer of hexamethyldisilazane (HMDS, Sigma, USA). The HMDS‐coated substrate was then spun with positive photoresist (RuiHong, China). Next, the substrate was exposed to UV light using a chrome‐based photomask, and then dissolved and removed using a developing solution. In the following step, HMDS exposed by photoresist was removed by oxygen plasma treatment for 2 min. Residual photoresist was removed by treating the substrate with acetone, and rinsing with isopropanol and deionized water. Then, the substrate was coated with 200 µg mL^−1^ poly‐l‐lysine‐poly (ethylene glycol)‐silane (pLL‐PEG) (SuSoS, Switzerland) solution for 1 h. For only short‐term limitations, 2 µg mL^−1^ PLL‐PEG was substituted for 1 h. Subsequently, the substrates were coated with 20 µg mL^−1^ fibronectin (FN, BD, USA) for 1 h. After removing the FN solution, NIH3T3 cells were plated on the substrates at 37 °C for 15 min at a seeding density of ≈2 × 10^5^ cells cm^−2^. By rinsing with culture medium, nonadherent cells were removed. Finally, the adherent cells were treated with 10 µg mL^−1^ mitomycin and 1 µg mL^−1^ Hoechst 33342 for 1 h.

### Time‐Lapse Living Cell Imaging

Cells confined in micropatterned substrates were cultured in an incubator (INUB‐ZILCSGH‐F1, Tokai Hit, Japan) mounted on an inverted optical differential interference contrast microscope (Ti‐E, Nikon, Japan), at 37 °C with 5% CO_2_ under humidified atmosphere. In the medium stirring experiment, the culture medium was slowly aspirated from the dish and quickly mixed it in a sterile tube, then gently added it back into the culture dish. Images were captured by an sCMOS (ORCA‐flash4.0, Hamamatsu, Japan), using multichannel mode to simultaneously obtain bright field and fluorescence data. The system was controlled with Micro‐Manager 1.4 software (NIH, USA).

### Modified Kernel Density Estimation Algorithm

First, the nuclei were intelligently identified by the ImageJ plugin *StarDist* combined with manual adjustments. The cell localization is determined by the mass center of nucleus. Cell density distribution was then estimated using the Kernel density estimation algorithm:

(5) $$p_{0}\left(r\right)=\frac{1}{n}\sum_{i=1}^{n}K\left(\frac{r-r_{i}}{h}\right),\;\mathrm{where}\;K\left(r\right)=\frac{1}{2\pi }\exp\left(-\frac{1}{2}{\left|r\right|}^{2}\right)$$

where **r** is the spatial position, **r**_*i*_ is the center of cell, *n* is the cell number, *K*(*x*) is the Kernel function estimated as a Gaussian distribution, *h* is the optimal bandwidth.^[^ ^64^ ^]^ Since cells were confined within the pattern, low‐density artifacts near the boundary must be removed by a boundary correction

(6) $$p^{\prime }\left(r\right)=\frac{p_{0}\left(r\right)}{\underset{S}{\;\int \int \;}K\left(\frac{r-r^{\prime }}{h}\right)dr^{\prime }}$$

where *S* denotes the restricted pattern region. The average density is normalized to 1:

(7) $$p\left(r\right)=\frac{p^{\prime }\left(r\right)}{\frac{1}{S}\underset{S}{\;\int \int \;}p^{\prime }\left(r^{\prime }\right)dr^{\prime }}$$

After obtaining the relative cell density distribution *p*(**r**), it was azimuthally collapsed with a ring‐width of Δ*r* = 20 µm to derive radial profiles of cell density *P*(*r*), defined as 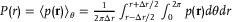, where (*r*, *θ*) are polar coordinates with the circle center as origin.

### Particle Image Velocimetry (PIV)

The velocity field was calculated using a custom algorithm based on MatPIV software package in MATLAB (mathworks, USA), with a 16 × 16 µm^2^ interrogation window (24 pixels window size) and 50% overlap. The time interval was 5 min between two consecutively analyzed frames.

### Kymograph of Radial Order Parameter and Radial Ordered Wavefront

First, the circle was spatially divided into concentric rings with 25 µm ring‐width. Next, the radial order parameter *q*(**r**, *T*) was averaged in each ring to obtain its radial profile. Similar steps were performed every 3 h to obtain a heatmap representing the spatiotemporal evolution of radial order parameter. Afterward, the kymograph was transformed into a binarized plot with the threshold *q* = 0.2. There is a significant dividing line in the binarized plot, which looks like a wavefront. the points on the dividing line were linearly fitted, and the reciprocal of the slope of the fitted straight line is defined as the speed of this radial ordered wavefront (*V_w_*).

### The Developed Mechanical Model in 2‐D Spatial Constraint

According to the experimental system, the model is established in a 2‐D domain, with two independent variables: cell density *n*(**r**, *t*) and concentration of ECM proteins *c*(**r**, *t*). Based on two‐coupled nonlinear partial differential equations, it describes cell migration driven by ECM force and cell diffusion, as well as the production, and degradation of ECM proteins. These equations are written in dimensionless form:

(8) $$\left\{\begin{array}{c} \frac{\partial n}{\partial t}=\Delta n-\nabla \cdot \left(n\cdot u\right)\\ \frac{\partial c}{\partial t}=s\left(\frac{n}{1+n}-c\right) \end{array}\right.$$

where *s* is a scaling factor. In line with experiment, cell proliferation is not considered in this model. The term *n*/(1+*n*) is a simple Michaelis‐Menten kinetics,^[^ ^65^ ^]^ it describes the saturation effect of the rate at which cells produce ECM proteins. **u**(**r**, *t*) is the velocity derived from ECM force, decided by the follow equation:

(9) $$u\left(r,t\right)=\alpha \;\int \int \;\frac{{\left[c\left(r,t\right)c\left(r^{\prime },t\right)\right]}^{1/2}\cdot s^{3/2}}{s^{2}{\left|r-r^{\prime }\right|}^{4}+{L_{u}}^{4}}\cdot \frac{r-r^{\prime }}{\left|r-r^{\prime }\right|}dr^{\prime }$$

where *α* measures the strength of the ECM force, *L_u_* is characteristic length. The original form of the Equations (8) and (9) along with their dimensionless quantification are shown in *Supporting information*.

Corresponding to experimental 2‐D spatial restriction, the cells can only exist in a circular region *Ω*, with boundary condition:

(10) $$J\cdot n=\left(\nabla n-nu\right)\cdot n=0\;\;\mathrm{for}\;\;r\in \partial \Omega ,$$

where **J** is cell flow, *Ω* is the pattern region. **n** is normal vector of pattern boundary ∂*Ω*. To quantify the equivalent velocity of cells, the cellular velocity field $V=\frac{J}{n}=\frac{\nabla n}{n}-u$ was defined, which is the superposition of $V_{d}=\frac{\nabla n}{n}$ and **V**_**c**_ = **u** due to cell diffusion and ECM force. In addition, radial velocity *V*(*r*) is defined as the radial component of **V**, namely *V*(*r*) = 〈[**V**]_*r*_〉_*θ*._

It was set that cells were uniformly distributed within the pattern at the starting time point, with the density defined as 1. Besides, the initial system did not contain ECM proteins, with initial condition setting as:

(11) $$n\left(r,0\right)=\left\{\begin{array}{c} 1,\;\;\mathrm{for}\;\;r\in \Omega \\ 0,\;\;\mathrm{else} \end{array}\right.,c\left(r,0\right)=0$$

An order of magnitude changes in parameters *α* and *s* dramatically affect the degree of aggregation, as shown by the results of density heterogeneity *η*, but they do not alter the tendency of cell migration toward the center (Figure S6, Supporting Information). Proper parameter values were chosen to make the simulation results reasonably close to experimental data, such as the density heterogeneity *η* (≈0.2), ensuring no significant differences in numerical magnitude.

### Statistical Analysis

All data are presented as mean ± standard error of mean (SEM) from at least three independent experiments. The statistical comparison between the two groups was carried out using Student's t‐test (OriginPro 2022, OriginLab). Statistical significance was defined as ^*^ *p* < 0.05, ^**^ *p* < 0.01, ^***^ *p* < 0.001, ^****^ *p* < 0.0001, NS, not significant (*p* > 0.05).

## Conflict of Interest

The authors declare no conflict of interest.

## Author Contributions

L.P. conceived the research and was in charge of the overall direction. L.P. and H.D. designed the experiments. H.D. and X.M. performed the experiments. H.D. performed computational modeling. H.D., F.H., X.M., and J.Y. analyzed the data. F.H. and J.Y. contributed to the interpretation of the results. F.H., H.D., and L.P. wrote the manuscript. L.P. and J.X. supervised the work.

## Supporting information

Supporting Information

Supplemental Movie1

Supplemental Movie2

Supplemental Movie3

Supplemental Movie4

Supplemental Movie5

Supplemental Movie6

Supplemental Movie7

## Data Availability

The data that support the findings of this study are available from the corresponding author upon reasonable request.
