# Supplementary material for: Collective Cell Radial Ordered Migration in Spatial Confinement
Source: Adv Sci (Weinh). 2024 Mar 23;11(20):2307487. doi: 10.1002/advs.202307487 (PMC11132034; doi:10.1002/advs.202307487)
Supplement: Supplementary file 1 — Supporting Information [file ADVS-11-2307487-s002.pdf]

ADVANCED SCIENCE
Open Access

Supporting Information

for *Adv. Sci.*, DOI 10.1002/advs.202307487

Collective Cell Radial Ordered Migration in Spatial Confinement

Hao Dong, Fen Hu, Xuehe Ma, Jianyu Yang, Leiting Pan and Jingjun Xu*

Supporting Information

Collective cell radial ordered migration in spatial confinement

Hao Dong[1], Fen Hu[1], Xuehe Ma[1], Jianyu Yang[1], Leiting Pan[1,2,3,4]*, Jingjun Xu[1,3]

[1]The Key Laboratory of Weak-Light Nonlinear Photonics of Education Ministry, School of Physics and TEDA Institute of Applied Physics, Nankai University, Tianjin 300071, China

[2]State Key Laboratory of Medicinal Chemical Biology, Frontiers Science Center for Cell Responses, College of Life Sciences, Nankai University, Tianjin 300071, China

[3]Shenzhen Research Institute of Nankai University, Shenzhen, Guangdong 518083, China

[4]Collaborative Innovation Center of Extreme Optics, Shanxi University, Taiyuan, Shanxi 030006, China.

*Correspondence: plt@nankai.edu.cn

Supplementary Experimental Section/Methods

The original form of equations in the developed mechanical model: Our developed mechanical model describes the evolution of cell density $n(\boldsymbol{r},t)$ and concentration of ECM proteins $c(\boldsymbol{r},t)$ in 2-D spatial constraint. The origin equation is written as followed:

$$\begin{cases} \dfrac{\partial n}{\partial t} = D_n \Delta n + \nabla \cdot (n \cdot \boldsymbol{u}), \\ \dfrac{\partial c}{\partial t} = \dfrac{Sn}{(\beta + n)} - \gamma c \end{cases} \tag{S1}$$

where D_n is the cell diffusion coefficient, the parameters S and γ are the maximum rate of ECM proteins secretion and the rate of degradation. $\boldsymbol{u}(\boldsymbol{r},t)$ is the velocity derived from ECM force, decided by the force balance equation

$$\iint \frac{\kappa [c(\boldsymbol{r},t)c(\boldsymbol{r}',t)]^{1/2}}{|\boldsymbol{r}-\boldsymbol{r}'|^4 + L_u{}^4} \cdot \frac{\boldsymbol{r}-\boldsymbol{r}'}{|\boldsymbol{r}-\boldsymbol{r}'|} d\boldsymbol{r}' = \mu \cdot \boldsymbol{u}(\boldsymbol{r},t) \tag{S2}$$

Here, we assume that the tension exerted by a cell on surrounding cells through the ECM is directly proportional to the average ECM proteins concentration $[c(\boldsymbol{r},t)c(\boldsymbol{r}',t)]^{1/2}$ and negatively correlated with the distance $|\boldsymbol{r}-\boldsymbol{r}'|$ between cells, where characteristic length L_u and $|\boldsymbol{r}-\boldsymbol{r}'|^4$ respectively represent short-range and long-range effects of ECM forces. The integral term on the left side of the equation (S2) signifies the resultant force from their surrounding cells, which is equivalent to the frictional force $\mu \cdot \boldsymbol{u}(\boldsymbol{r},t)$ originated from cell-substrate under overdamped conditions. κ is ECM force coefficient, μ is friction coefficient. The equations can be nondimensionalized by setting

$$x^* = \left(\frac{\gamma}{sD_n}\right)^{1/2} x, \quad t^* = \frac{\gamma t}{s}, \quad n^* = \frac{n}{\beta},$$
$$c^* = \frac{\gamma c}{S}, \quad \alpha^* = \frac{S}{\gamma^{1/2} {D_n}^{3/2}} \cdot \frac{\kappa}{\mu}, \quad {L_u}^* = \left(\frac{\gamma}{D_n}\right)^{1/2} L_u, \tag{S3}$$

where s is a scaling factor, an additional parameter added to conveniently change the spatiotemporal scale of the system. The equivalent size of the system is proportional to $s^{1/2}$. Using above equation and removing the asterisk for simplicity, the equation (S1) and (S2) become

$$\begin{cases} \dfrac{\partial n}{\partial t} = \Delta n - \nabla \cdot (n \cdot \boldsymbol{u}), \\ \dfrac{\partial c}{\partial t} = s\left(\dfrac{n}{1+n} - c\right) \end{cases} \tag{S4}$$

$$\boldsymbol{u}(\boldsymbol{r}, t) = \alpha \iint \frac{[c(\boldsymbol{r}, t) c(\boldsymbol{r}', t)]^{1/2} \cdot s^{3/2}}{s^2 |\boldsymbol{r} - \boldsymbol{r}'|^4 + {L_u}^4} \cdot \frac{\boldsymbol{r} - \boldsymbol{r}'}{|\boldsymbol{r} - \boldsymbol{r}'|} d\boldsymbol{r}' \tag{S5}$$

That is the equation used for our simulation.

Co-culture and staining experiment of MDCK and NIH3T3 cells: A PDMS slice with a width of 1000 μm was stuck to the center of glass substrate, forming two separate compartments. MDCK cells and NIH3T3 cells were planted in two compartments and attached to the glass substrate. Unattached cells were washed away, and then PDMS slice was gently removed. It created a 1000 μm gap, flanked by monolayers of MDCK and NIH3T3 cells, respectively. The sample were cultured in incubators, during which the cells on both sides migrated to the gap. Afterward, the sample was fixed with 4% paraformaldehyde before the cells on both sides converged. The sample was permeabilized with blocking solution (3% BSA in 0.05% Triton X-100) for 30 min. Cells were then incubated in rabbit anti-E-cadherin antibody (1:200 diluted in blocking solution) for 1 h,

washed with PBS and incubated in Alexa-647-labelled goat anti-rabbit antibody (1:200 diluted in blocking solution) for 40 min. Finally, the cells were incubated in 1 μg/ml Hoechst 33342 for 10 min.

Cell Potts model (CPM): CPM is a classical mathematical model used to simulate the movement and interactions of cells[s1,s2], as we previously used[s3]. The model is implemented on a discrete spatial grid. Each cell is depicted as an assembly of multiple neighboring grid sites. To simulate cell spreading, a random grid site tries to copy itself to a neighboring grid based on the Metropolis algorithm. The acceptance probability of a copy depends on the energy difference ΔH of the system after and before this copy. The energy H arises from cell surface mechanics and actin cytoskeleton. The energy of cell surface mechanics H_{CSM} is calculated by

$$H_{CSM} = \sum_{i,j} J_{\sigma_i,\sigma_j}(1-\delta_{\sigma_i,\sigma_j}) + \sum_{\sigma} \lambda_{Area}(a_\sigma - A_\sigma)^2 + \sum_{\sigma} \lambda_{Perimeter}(p_\sigma - P_\sigma)^2 \quad \text{(S6)}$$

where the first term accounts for the adhesion energy of cells, with J is the adhesion coefficient (a smaller value of J indicates stronger cell adhesion). The second and third terms describe the area constraint, and the perimeter constraint of cells respectively. Moreover, the actin cytoskeleton in the model provides energy for driving cell migration. The orientation of cell migration depends on the parameter of Max_{act}, which stands for actin activity. The larger Max_{act}, the more directional the cell movement is (from Brownian-like motion to ballistic-like motion). Further details of actin dynamics in the model can be found in previous literature[s4]. On the other hand, to simulate the geometric confinement in the experiment, we set up a circular adhesive region on the spatial grid and assign an extremely high energy barrier in non-adhesive regions. Removing this energy barrier will result in cell spreading from inside to outside of the pattern. The meanings and values of all parameters are shown in Table S2, Supporting Information.

Supplementary References

[S1] F. Graner, J. A. Glazier, *Phys. Rev. Lett.* **1992**, *69*, 2013.

[S2] R. Alert, X. Trepat, *Annu. Rev. Condens. Matter Phys.* **2020**, *11*, 77.

[S3] F. Xing, H. Dong, J. Yang, C. Fan, M. Hou, P. Zhang, F. Hu, J. Zhou, L. Chen, L. Pan, J. Xu, *Adv. Sci.* **2023**, *10*, e2301337.

[S4] I. Niculescu, J. Textor, R. J. de Boer, *PLOS Comput. Biol.* **2015**, *11*, e1004280.

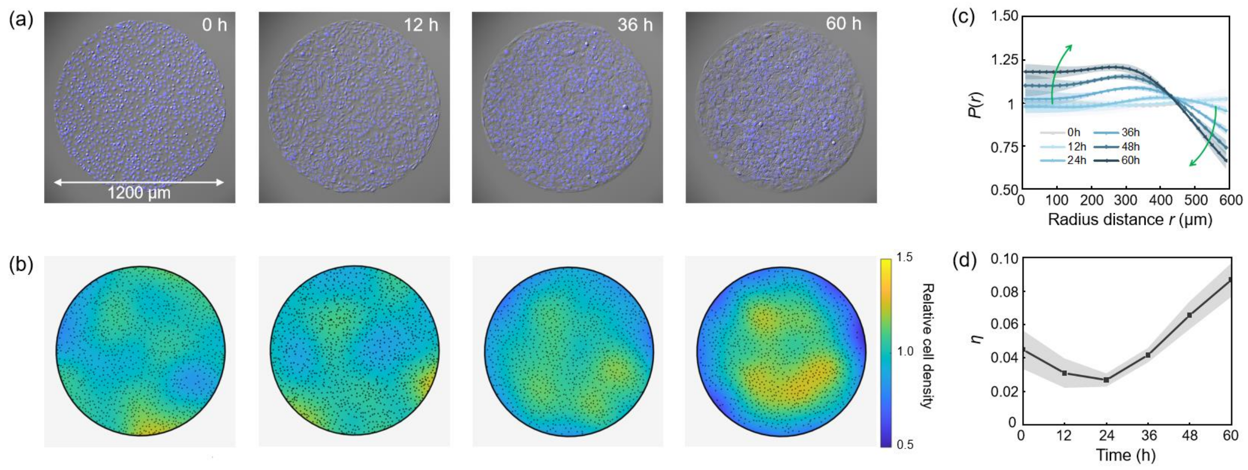

Figure S1. The collective centripetal migration of NIH3T3 cells confined in larger circular patterns. (a) Time evolution of NIH3T3 fibroblasts confined to 1200-μm circular patterns. Nuclei are marked in blue. (b) Heat maps of relative cell density $p(\boldsymbol{r})$. The black dots represent the mass center of nuclei, corresponding to (a). (c) Radial profiles of relative cell density $P(r)$ at 0, 12, 24, 36, 48, 60 h (n=3, mean ± SEM). (d) Temporal evolutions of cell density heterogeneity η (n=3, mean ± SEM).

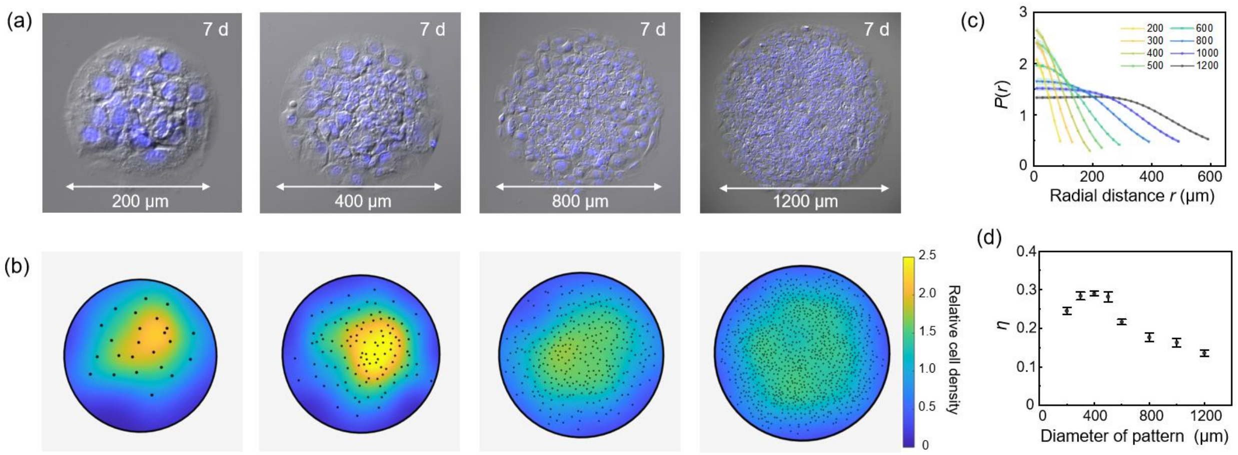

Figure S2. The collective centripetal migration of cells confined to different sizes of circular patterns. (a) Cells incubated for 7 days in circular patterns with varying diameters to ensure adequate steady cell density distribution. (b) Heat maps of relative cell density $p(\boldsymbol{r})$. The black dots represent the mass center of nuclei, corresponding to (a). (c-d) Radial profiles of relative cell density $P(r)$ (c) and density heterogeneity η (d) for different sizes of pattern after incubated for 7 days (n=12 for all sizes).

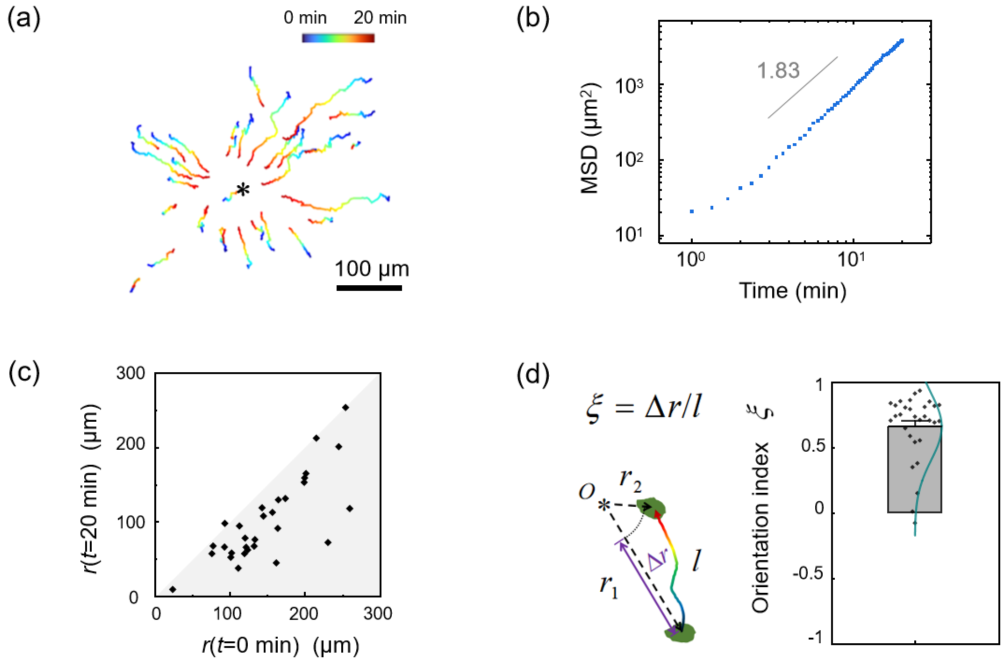

Figure S3. The motion characteristics of ADP-induced microglia chemotactic migration, an acknowledged strong-oriented migration process. (a) Trajectories of cells visualized in a time-coded color representation, ranging from blue to red. The asterisk represents the position of chemotactic source. (b) MSDs of cells with a scaling exponent α=1.83 (n=30). (c) The scatter plot showing the relation of radial positions of the same cell before and after the experiment. Pearson's correlation coefficient is ρ=0.680. Points in gray region represents cells that are closer to the center. (d) Diagram of orientation index ξ (left) and its statistical distributions of cells (ξ = 0.658 ± 0.047, n=30), fitted by a Gaussian profile (right).

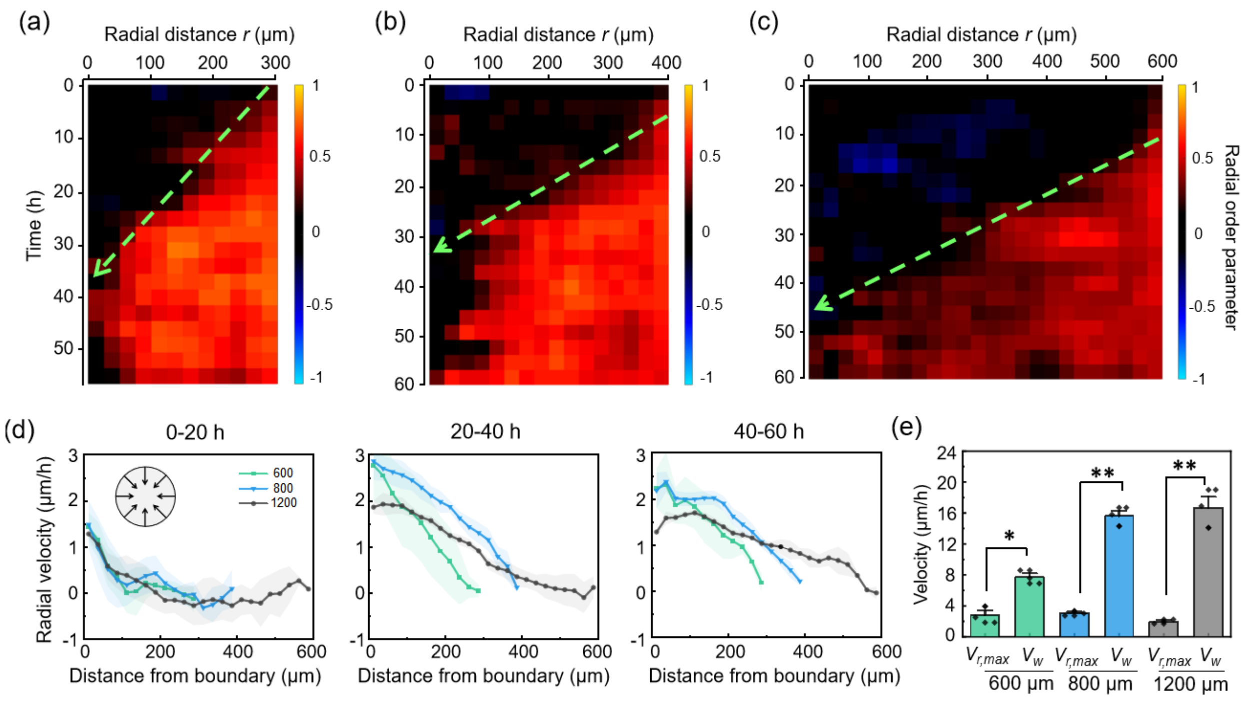

Figure S4. Spatiotemporal dynamics of collective cells in larger constrained patterns. (a-c) Kymographs of the radial order parameter for 600 μm (a), 800 μm (b), and 1200 μm (c) diameter circular islands. Dashed green lines indicate fitted dividing lines with a threshold q=0.20. (d) Profiles of radial velocity for 600 μm, 800 μm, and 1200 μm diameter circular islands (n=3 for all sizes, mean ± SEM). (e) Comparison between the maximum radial velocity $V_{r,\max}$ and the radial ordered wave speed V_w for cells confined in the three pattern sizes. (n=3 for all sizes, *P<0.05, **P<0.01, ***P<0.001, paired t-test).

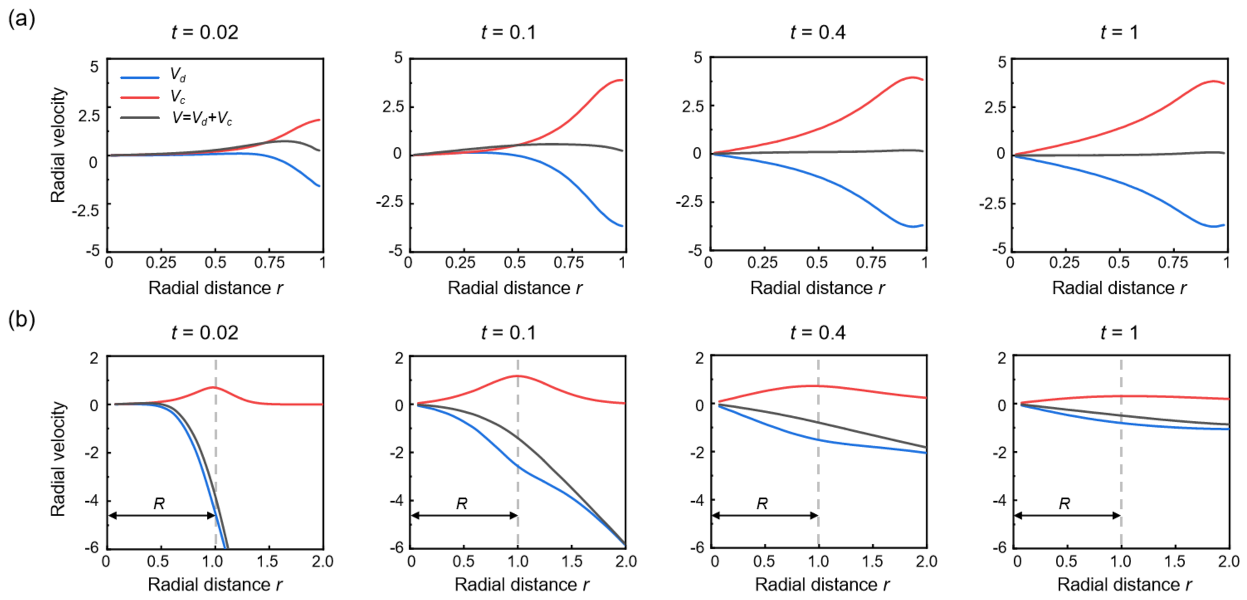

Figure S5. Simulated evolution of radial cellular velocity $V(r)$ (gray), as well as its diffusion component $V_d(r)$ (blue) and ECM force-dependent component $V_c(r)$ (red) at different time points with constant constraints (a) or removing constraints at $t > 0$ (b), corresponding to Fig. 4(d) and Fig. 4(e), respectively. A positive value means that the velocity direction is inward, and a negative value means that the velocity direction is outward. (α=1.8, s=16, L_μ=1 and R=1).

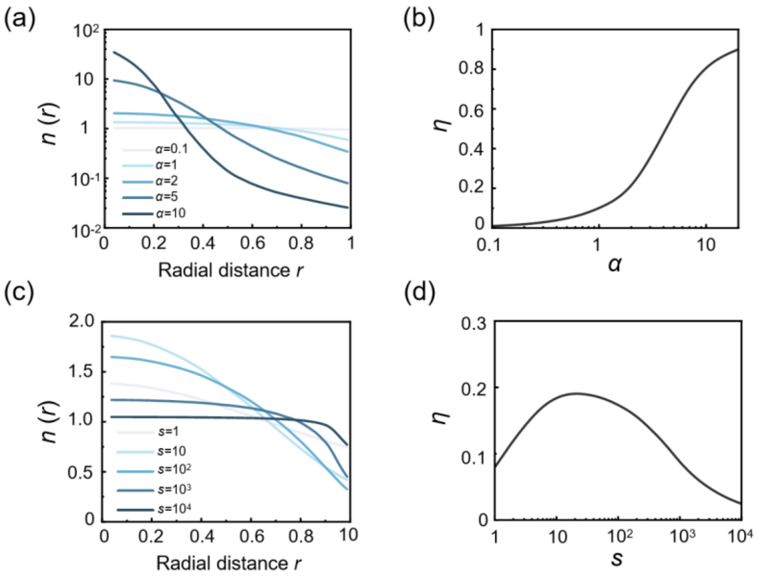

Figure S6. Sensitivity analysis for the parameters of the developed mechanical model in 2-D spatial constraint. (a-b) Simulation of radial cell density profiles $n(r)$ (a) and density heterogeneity η (b) with varying the value of α from 0.1 to 10 (s=16, L_μ=1, and R=1). (c-d) Simulation of radial cell density profiles $n(r)$ (c) and density heterogeneity η (d) with varying the value of s from 1 to 10000 (α=1.8, L_μ=1, and R=1).

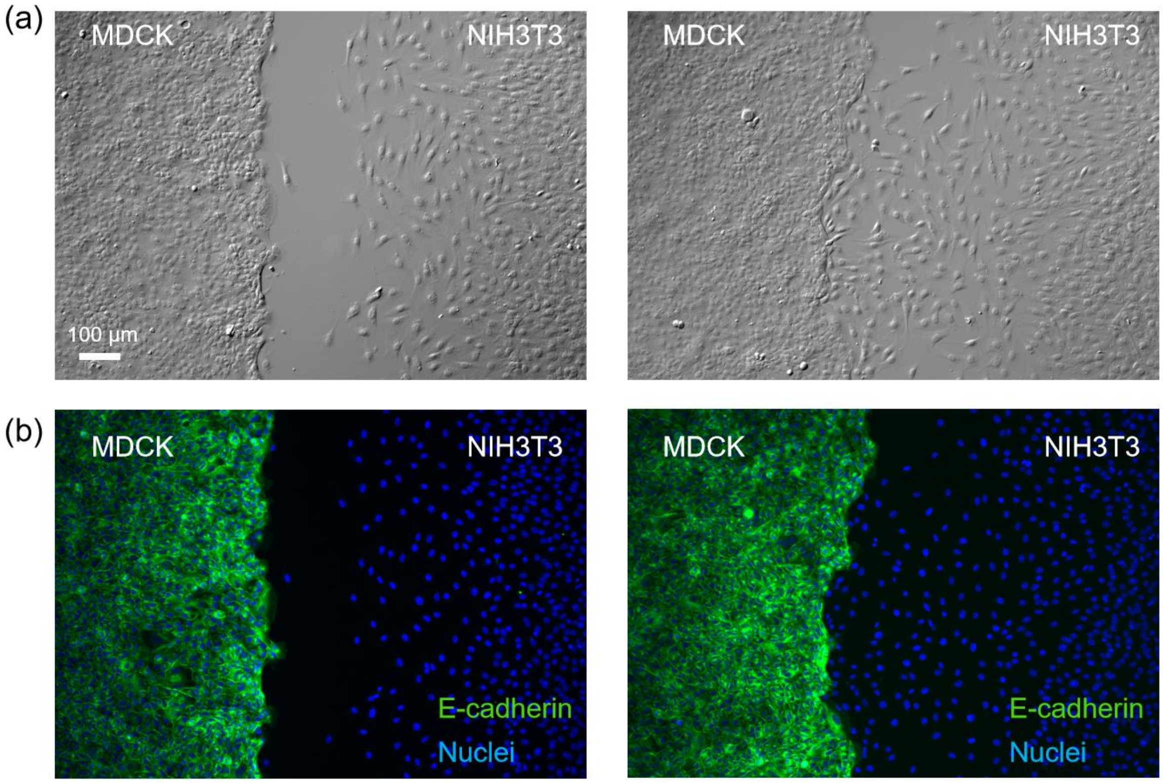

Figure S7. Typical fixed images of collective MDCK cells and NIH3T3 cells. (a) Bright field of cell migration of MDCK cells and NIH3T3 cells from both sides to the center. (b) The fluorescence staining images of E-cadherin (green) and nuclei (blue), corresponding to (a). It shows that E-cadherin is highly expressed in MDCK cells while weakly expressed in NIH3T3 cells.

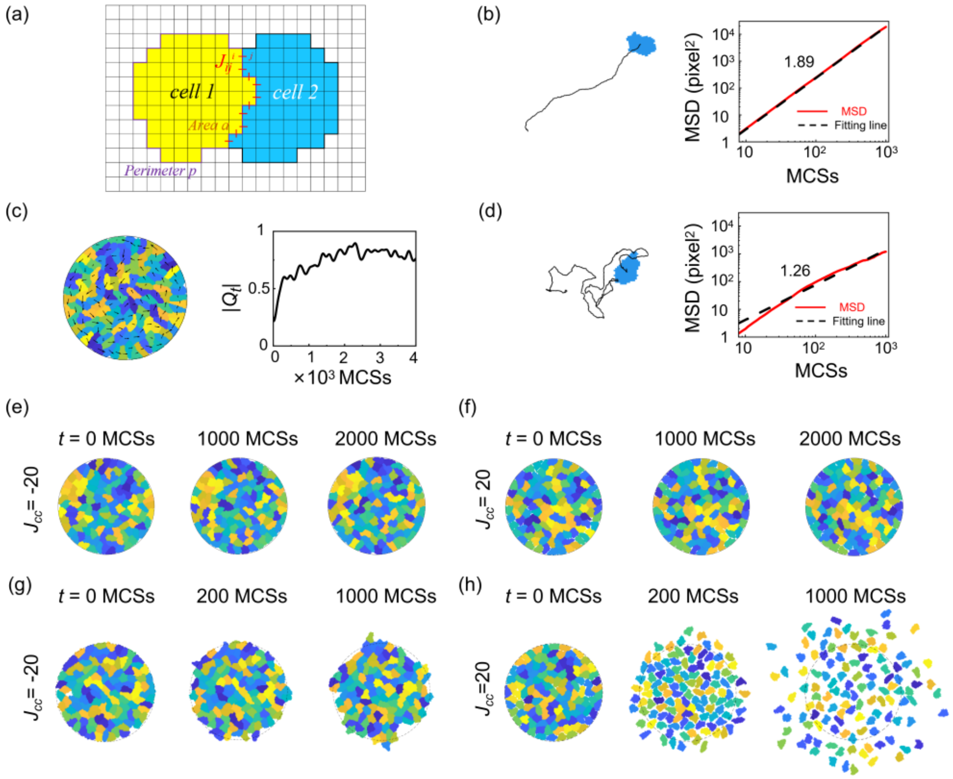

Figure S8. CPM simulations of collective cell migration in circular confinement. (a) Diagram of cell-cell adhesion and steric interaction in CPM. (b) The typical trajectory of ballistic-like cell motion and corresponding MSD curve (n=120), with a scaling exponent α=1.89 (Max_{act} =35). (c) Simulations of collective cell rotation in circular pattern and evolution of corresponding tangential order parameter $|Q_t|$ (Max_{act} =35, J_{cc} =-20). (d) The typical trajectory of Brownian-like cell motion and corresponding MSD curve (n=120), with a scaling exponent α=1.26 (Max_{act} =15). (e-f) Time evolution of the collective cells with strong adhesion (J_{cc} =-20) (e) and weak adhesion (J_{cc}=20) (f) conditions in circular confinement (Max_{act} =15). (g-h) Time evolution of collective cells with strong adhesion (J_{cc} =-20) (g) and weak adhesion (J_{cc} =20) (h) conditions after removing circular confinement at t=0 (Max_{act} =15). Cell colors are arbitrary for all conditions. MCSs, Monte Carlo steps. Other simulation parameters not mentioned are shown in Table S2, Supporting Information.

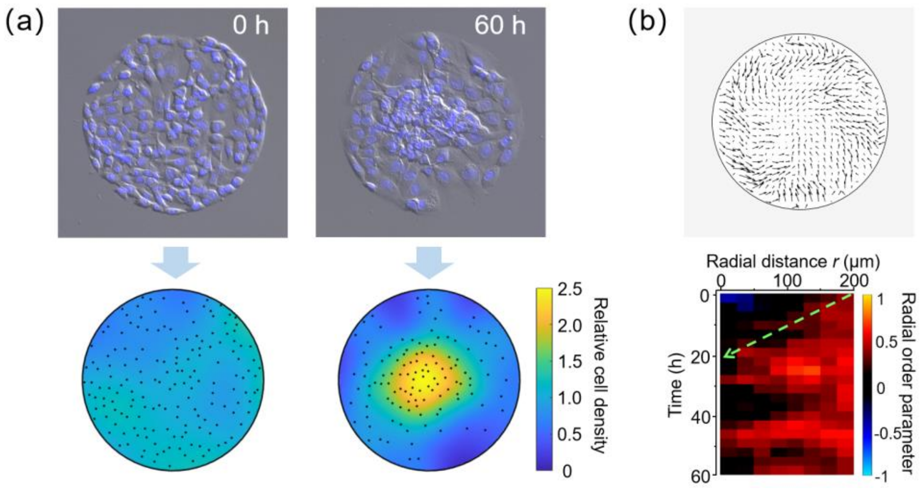

Figure S9. The collective migration of NIH3T3 cells in circular confinement with medium stirring. (a) Bright fields (up) and heat maps (down) of relative cell density $p(\mathbf{r})$ of NIH3T3 fibroblasts persistently confined to a 400-μm diameter circular pattern, with stirring the medium every ~2 h. The nuclei were labeled by Hoechst 33342 (blue). (b) Average velocity field maps (10-50 h) of collective cells (up) and kymograph of the radial order parameter (down), corresponding to (a).

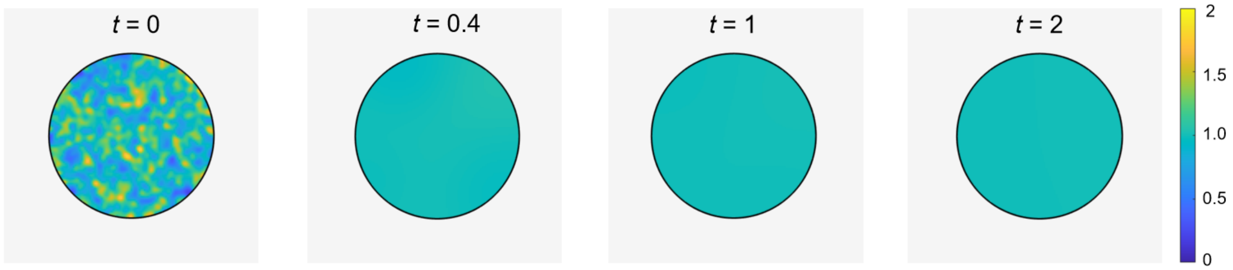

Figure S10. Simulations of collective cell behavior in circular restriction excluding endogenous ECM-associated plithotaxis of cells (α=0, s=16 and R=1). Cells are randomly distributed in the pattern at t=0.

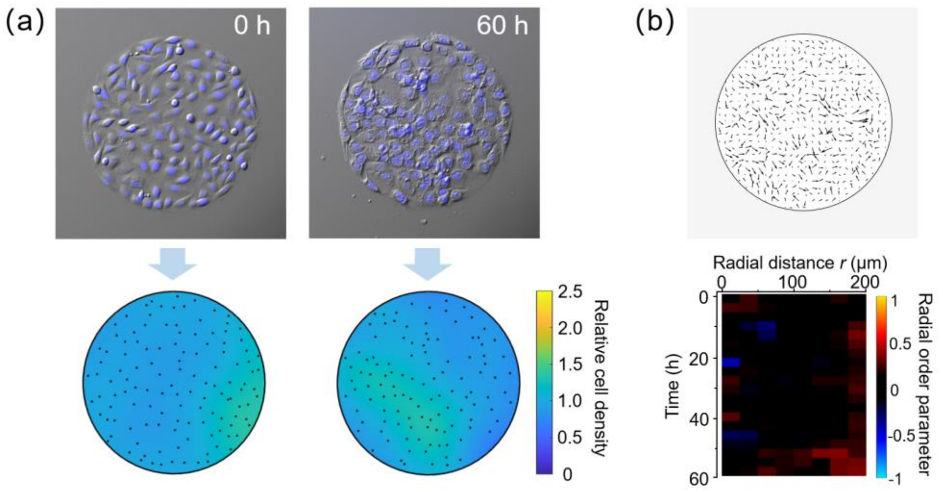

Figure S11. The collective migration of NIH3T3 cells confined to a 400-μm diameter circle pattern in Ca^{2+}-free condition. (a) Bright fields (up) and heat maps (down) of relative cell density $p(\boldsymbol{r})$ of NIH3T3 cultured in Ca^{2+}-free medium. The nuclei were labeled by Hoechst 33342 (blue). (b) Average velocity field maps (10-50 h) of collective cells (up) and kymograph of the radial order parameter (down), corresponding to (a).

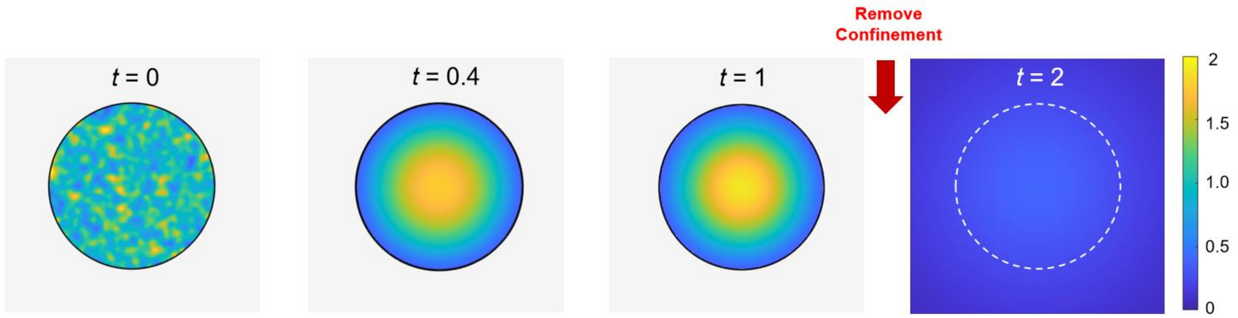

Figure S12. Simulations of collective cell behavior in circular restriction considering endogenous ECM-associated plithotaxis of cells. Cells are randomly distributed in the pattern at t=0 (α=1.8, s=16, L_μ=1 and R=1). Constraint was removed at t=1 with other parameters kept constant.

Table S1. Parameters used in the developed mechanical model in 2-D spatial constraint

Name	Meaning	Value
α	the strength of the ECM force	1
s	Size factor	16
L_u	characteristic length	1
R	Radius of circular pattern	1
d	Side length of the square pattern	1.5
R_p	Negative radius of indented-square boundary	1.5
R_o	Circle radius of the peanut-like patterns	0.5
d_o	The overlap distance between two circles in the peanut-like pattern	0.07

Table S2. Parameters used in CPM

Name	Meaning	Value
T	System temperature	20
J_{cc}	Adhesion coefficient across cell-cell interfaces	20 for weak adhension<br>-20 for strong adhension
J_{CM}	Adhesion coefficient across cell-matrix interfaces	0
λ_{Area}	Area stiffness	4
$\lambda_{Perimeter}$	Perimeter stiffness	2
A_{σ}	Target area	201
P_{σ}	Target Perimeter	170
λ_{act}	Maximum energy contribution of actin	100
$(V_{act})_{\max}$	Maximum actin activity	15 for Brownian-like motion<br>35 for ballistic-like motion
N	Number of cells	120

Movie S1: Time-lapse video of NIH3T3 fibroblasts confined to a 400-μm diameter circle pattern. The nuclei were labeled by Hoechst 33342 (blue).

Movie S2: Time-lapse video of NIH3T3 fibroblasts confined to a 1200-μm diameter circle pattern. The nuclei were labeled by Hoechst 33342 (blue).

Movie S3: Time-lapse video of ADP-induced microglia chemotactic migration.

Movie S4: Simulated evolution of cell density distribution in circular constraint by the developed mechanical model.

Movie S5: Time evolution of NIH3T3 fibroblasts confined to a square (left), indented-square (middle), and peanut-like (right) patterns.

Movie S6: Time-lapse video of NIH3T3 fibroblasts confined to a 400-μm diameter circle pattern with medium stirring every ~2 h.

Movie S7: Time-lapse video of NIH3T3 fibroblasts confined to a 400-μm diameter circle pattern in Ca^{2+}-free condition.
